# Stratifying non-small cell lung cancer patients using an inverse of the treatment decision rules: validation using electronic health records with application to an administrative database

**DOI:** 10.1186/s12911-022-02088-x

**Published:** 2023-01-06

**Authors:** Min-Hyung Kim, Sojung Park, Yu Rang Park, Wonjun Ji, Seul-Gi Kim, Minji Choo, Seung-Sik Hwang, Jae Cheol Lee, Hyeong Ryul Kim, Chang-Min Choi

**Affiliations:** 1 Department of Epidemiology, Harvard T.H. Chan School of Public Health, Harvard University, Boston, MA USA; 2 Department of Preventive Medicine and Family Medicine, College of Medicine, Seoul National University, Seoul, Republic of Korea; 3 Department of Respiratory and Critical Care Medicine, College of Medicine, Ewha Womans University Medical Center, Seoul, Republic of Korea; 4 Department of Biomedical System Informatics, Yonsei University College of Medicine, Seoul, Republic of Korea; 5 Department of Respiratory and Critical Care Medicine, Asan Medical Center, University of Ulsan College of Medicine, Seoul, Republic of Korea; 6 Department of Epidemiology, Graduate School of Public Health, Seoul National University, Seoul, Republic of Korea; 7 Department of Oncology, College of Medicine, Asan Medical Center, University of Ulsan, Seoul, Republic of Korea; 8 Department of Thoracic and Cardiovascular Surgery, College of Medicine, Asan Medical Center, University of Ulsan, Seoul, Republic of Korea; 9 Department of Pulmonary and Critical Care Medicine, Department of Oncology, Asan Medical Center, University of Ulsan College of Medicine, 88 Olympic-Ro 43-Gil, Songpa–gu, Seoul, 05505 South Korea

**Keywords:** Treatment decision rules, TNM Stage, Non-small cell lung cancer, Electronic health record, Administrative database

## Abstract

**Background:**

To validate a stratification method using an inverse of treatment decision rules that can classify non-small cell lung cancer (NSCLC) patients in real-world treatment records.

**Methods:**

(1) To validate the index classifier against the TNM 7th edition, we analyzed electronic health records of NSCLC patients diagnosed from 2011 to 2015 in a tertiary referral hospital in Seoul, Korea. Predictive accuracy, stage-specific sensitivity, specificity, positive predictive value, negative predictive value, F1 score, and c-statistic were measured. (2) To apply the index classifier in an administrative database, we analyzed NSCLC patients in Korean National Health Insurance Database, 2002–2013. Differential survival rates among the classes were examined with the log-rank test, and class-specific survival rates were compared with the reference survival rates.

**Results:**

(1) In the validation study (N = 1375), the overall accuracy was 93.8% (95% CI: 92.5–95.0%). Stage-specific c-statistic was the highest for stage I (0.97, 95% CI: 0.96–0.98) and the lowest for stage III (0.82, 95% CI: 0.77–0.87). (2) In the application study (N = 71,593), the index classifier showed a tendency for differentiating survival probabilities among classes. Compared to the reference TNM survival rates, the index classification under-estimated the survival probability for stages IA, IIIB, and IV, and over-estimated it for stages IIA and IIB.

**Conclusion:**

The inverse of the treatment decision rules has a potential to supplement a routinely collected database with information encoded in the treatment decision rules to classify NSCLC patients. It requires further validation and replication in multiple clinical settings.

**Supplementary Information:**

The online version contains supplementary material available at 10.1186/s12911-022-02088-x.

## Background

Lung cancer is one of the most common cancers and is the leading cause of cancer death worldwide [1, 2]. Stratification of lung cancer phenotypes is an essential step for treatment decision making, and TNM classification has an important role for therapeutic and prognostic guidance [3, 4]. As TNM staging is based on numeric dimensions (i.e., tumor size and location) and multinomial dimensions (i.e., invasion of lymph nodes and major organs), the TNM classification method projects the original feature space into a few tens of categories [5–7].

Increasingly, routinely collected administrative healthcare databases provide new opportunities in conducting large-scale analysis of actual clinical practice data in real-world settings [8–11]. However, the routinely collected databases tend to include only codified data elements with existing ontology, and they are prone to misclassification and missing information [12]. Therefore, efficient method to identify decision bases are necessary for studying large scale databases.

Observed, or revealed, decision actions can be used to estimate the information that the decision-makers used [13, 14]. The current study validated a stratification method for non-small lung cancer (NSCLC) patients based on an inverse of the treatment decision rules, which can be applied to codified data elements in administrative databases [11]. We validated the index classifier from two aspects. First, we examined the predictive performance when the index classification was compared to the TNM stage in the electronic health records (EHRs). Second, we examined whether the index classification shows differential survival rates when applied to a population-based administrative database.

## Methods

### The index classification: an inverse of the treatment decision function

The index classification is based on an inverse of the treatment decision rules given in the recommended treatment regimens in the National Comprehensive Cancer Network (NCCN) guidelines [4, 11]. The inverse of the stage-based treatment decision rules maps from the treatment regimens into patients' status. (Additional file 1: Tables S1, S2 and Methods 1). We only considered a subdomain of treatment patterns that are invertible and had sufficient samples. Seven potential categories for the inverse of the treatment decision function are shown in Table 1. Note that the inverse of the stage-based treatment decision function is pre-specified based on clinical knowledge, without any reference to the validation data.

**Table 1:** Potential categories for the inverse function of the treatment decision

Treatment pattern^†^				Corresponding TNM classification (7th edition)
1	Surgical resection			IA, IB, IIA
2	Surgical resection	Adjuvant CTx		(IIA,) IIB
3	Surgical resection	Adjuvant CTx	Adjuvant RTx	IIIA
4	Surgical resection	Adjuvant RTx	Adjuvant CTx	
5	Neoadjuvant CTx	Surgical resection		
6	Concurrent chemoradiation therapy			(IIIA,) IIIB
7	Chemotherapy			IV

*CTx*, Chemotherapy, *CCRT*, concurrent chemoradiation therapy, *RTx*, Radiotherapy

^†^We only considered a subdomain of treatment patterns that are invertible and have sufficient samples, ignoring stage IIA in the class 2 and stage IIIA in the class 6. Also, note that Stage IA, IB, and IIA can have the same treatment pattern

### Validation study for the index classification using electronic health records

We retrospectively validated the index classifier against the reference standard staging information in the EHRs of Asan Medical Center (AMC), a tertiary referral hospital in Seoul, Korea. The study protocol using EHR data was approved by the AMC, and all methods were performed in accordance with the relevant guidelines and regulations. We adopted the estimated sample size of 108 for each category for testing the accuracy of a single modality to detect a pre-specified area under the receiver operating characteristic curve value of 0.8 against a null value of 0.7 with a 95% confidence level and 80% power [15]. We identified a consecutive series of patients who were newly diagnosed with NSCLC and staged between 2011 and 2015, covered under the national health insurance, and received cancer treatment in the hospital with their last visit more than 180 days from the end of observation. We included those aged between 20 and 75 without a preexisting non-pulmonary cancer or a cardiopulmonary comorbidity, who may have been treated according to the treatment guidelines (Additional file 1: Methods 2) [16]. We excluded cases for which complete information for the index classification were not available: initially diagnosed at an external hospital, transferred to an external hospital, missing stage, erroneous stage identified during the manual chart review process, or received no treatment. In the sensitivity analysis, those with cardiopulmonary comorbidities were included in addition to the participants in the main analysis.

The reference stage, which was based on the TNM 7th edition [5] during the study period, was assigned by manual chart review for all of the study participants by a clinician who was blinded to the modeling to minimize potential bias. The stage was then inferred by a researcher who was blinded to the reference standard staging information, using the diagnosis codes, procedure codes, and medication codes.

Overall accuracy was calculated as the proportion of cases with correctly classified stages among the entire study population (Additional file 1: Methods 3). Stage-specific sensitivity, specificity, positive predictive value (PPV), negative predictive value (NPV), F1 score, and c-statistic were calculated by contrasting each stage with alternative stages [17–19]. The 95% confidence intervals for the performance metrics were estimated with 1000 bootstrap resamples.

### Stage-specific survival analysis applying the index classification in a population-based administrative database

We applied the index classifier to Korean National Health Insurance database, 2002–2013, retrospectively [20, 21]. We had access to treatment and survival information for the entire cohort of NSCLC patients and to the full claims records for a 2% random sample. We identified a consecutive series of all patients with the diagnosis code for lung cancer with at least one claim code for the treatment of NSCLC between 2004 and 2013. Patients with claim codes for lung cancer between 2002 and 2003 were excluded for a washout period, as they may have been diagnosed with lung cancer before 2002. We considered overall survival using the linked records in the national death registry with any cause. Class-specific survival rates were compared to the reference survival rates in the TNM 7th edition [22].

The proportional hazard assumption was rejected from the independency test for Schoenfeld residuals against time (Additional file 1: Methods 4) [23]. The survival function was estimated with the non-parametric Kaplan–Meier method, and the log-rank test was used to test the null hypothesis of no difference in the survival between groups. Pairwise log-rank tests were performed to explore significantly different survival curves, and the significance level of 0.05 was adjusted with Bonferroni methods. The 95% confidence intervals for the survival estimates were computed with the normal approximation. Analyses were performed using R statistical software version 3.5.3 (R Foundation for Statistical Computing) and SAS version 9.4 (SAS Institute Inc., NC, USA).

We considered the validation study as the main study, and this report follows the STARD reporting guideline for diagnostic studies [24, 25].

## Results

### Validation study for the index classification using the electronic health records

**Fig. 1:**
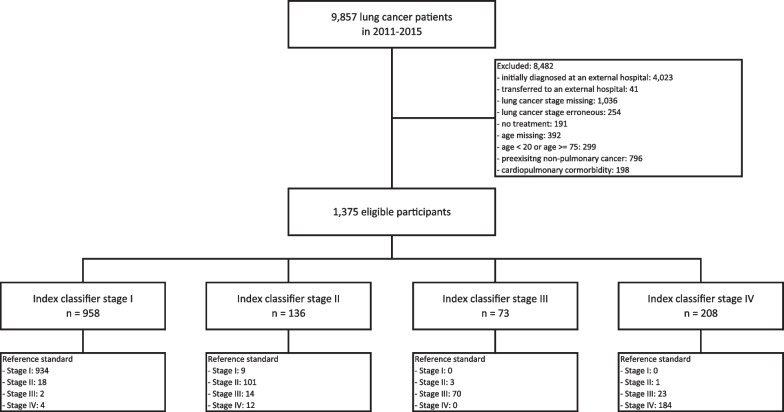
Population selection diagram of the validation study. To validate the index classifier against the TNM 7th edition, a retrospective study was conducted with electronic health records (EHRs) of NSCLC patients in a tertiary referral hospital in Seoul, Korea. We identified a consecutive series of all patients who were newly diagnosed with NSCLC and staged between 2011 and 2015, covered under the national health insurance, and received cancer treatment in the hospital with their last visit more than 180 days from the end of observation. We excluded cases initially diagnosed at an external hospital, transferred to an external hospital, missing stage information, erroneous stage identified during the manual chart review process, received no treatment, missing age information, age of less than 20 years or more than 75 years, preexisting non-pulmonary cancer, or cardiopulmonary comorbidity (eMethods 2)

The study population for the validation study consisted of 1375 NSCLC patients with a mean age of 60.3 (sd = 9.3) and a male-to-female ratio of 1.86. The population selection diagram for the validation study is shown in Fig. 1. The stage distribution was 68.6%, 8.9%, 7.9%, and 14.5% for stage I, II, III, and IV, respectively.

In the validation study, the overall accuracy of the index classification was 93.8% (95% CI: 92.5–95.0%). Stage I showed the highest stage-specific sensitivity (0.99, 95% CI: 0.98–0.99), PPV (0.97, 95% CI: 0.97–0.98), F1 score (0.98, 95% CI: 0.98–0.99), and c-statistic (0.97, 95% CI: 0.96–0.98), while specificity was highest for stage III (0.99, 95% CI: 0.99–0.99), and NPV was highest for stage IV (0.99, 95% CI: 0.98–0.99) (Table 2). On the other hand, stage-specific sensitivity (0.64, 95% CI: 0.55–0.74), F1 score (0.77, 95% CI: 0.70–0.84), and c-statistic (0.82, 95% CI: 0.77–0.87) were lowest for stage III, and PPV was the lowest for stage II (0.74, 95% CI: 0.67–0.81). A confusion matrix comparing the true stage and the inferred stage in the validation study is shown in Additional file 1: Table S3.

**Table 2:** Stage-specific predictive performance metric of the index classifier against the reference standard (TNM stage) (N = 1375)

Metric (95% CI)	I	II	III	IV
Sensitivity	0.99 (0.98, 0.99)	0.82 (0.75, 0.89)	0.64 (0.55, 0.74)	0.92 (0.88, 0.95)
Specificity	0.94 (0.92, 0.96)	0.97 (0.96, 0.98)	0.99 (0.99, 0.99)	0.98 (0.97, 0.99)
Positive predictive value	0.97 (0.97, 0.98)	0.74 (0.67, 0.81)	0.96 (0.91, 0.99)	0.88 (0.84, 0.93)
Negative predictive value	0.98 (0.96, 0.99)	0.98 (0.97, 0.99)	0.97 (0.96, 0.98)	0.99 (0.98, 0.99)
F1 score	0.98 (0.98, 0.99)	0.78 (0.72, 0.83)	0.77 (0.70, 0.84)	0.90 (0.87, 0.93)
c-statistic	0.97 (0.96, 0.98)	0.90 (0.86, 0.93)	0.82 (0.77, 0.87)	0.95 (0.93, 0.97)

In the sensitivity analysis, 198 NSCLC patients with cardiopulmonary comorbidity were included in the analysis (N = 1573). The overall accuracy of the index classification was 93.5% (95% CI: 92.3–94.7%). Sensitivity, specificity, PPV, NPV, and c-statistic were all within the confidence limits of the main analysis. Results for the sensitivity analysis are shown in Additional file 1: Tables S4, S5.

### Stage-specific survival analysis applying the index classification in a population-based administrative database

The study population for the application study consisted of 71,593 NSCLC patients with a mean age of 64.3 (sd = 10.2) and a male-to-female ratio of 72:28. Among a total of 166,203 patients who had a diagnosis code and at least one procedure or medication code for the treatment of NSCLC in the national health insurance database, 71,593 patients were eligible for analysis. Of these patients, 32.9% (23,571/71,593) received chemotherapy alone, 29.9% (21,378/71,593) received chemotherapy and radiotherapy, and 24.5% (17,543/71,593) underwent surgical resection alone.

**Fig. 2:**
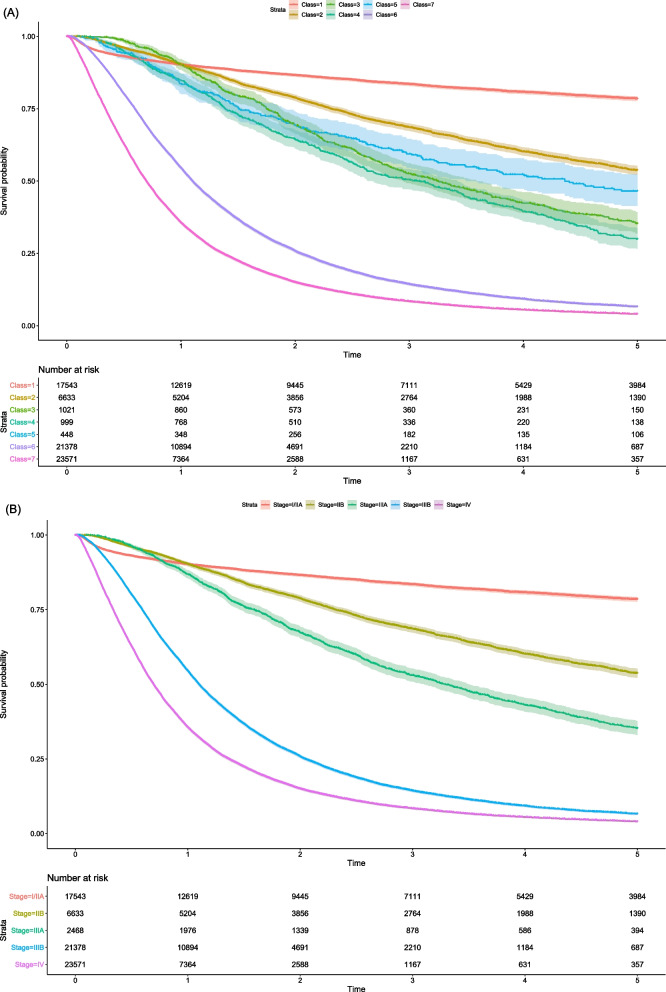
Class-specific (**A**) and stage-specific (**B**) survival curves with 95% confidence intervals and risk tables. **A** The index classification using an inverse of treatment decision function was applied to NSCLC cancer patients in Korean National Health Insurance database (N = 71,593), and class-specific survival function was estimated with the non-parametric Kaplan–Meier method. **B** The cancer stages (TNM 7th edition) were inferred from the index classification, and stage-specific survival function was estimated with the non-parametric Kaplan–Meier method. Stage I and IIA are aggregated in the survival curve because the index classification cannot distinguish them

When applying the index classifier for NSCLC patients, the independency test for Schoenfeld residuals against time rejected the proportional hazard assumption (*p* = 0.043). The global null hypothesis of no difference in the survival rate was rejected using the log-rank test (*p* < 0.001). Pairwise log-rank tests corrected with Bonferroni methods rejected most null hypotheses, except those comparing class 2 vs. class 5, and class 3 vs. class 4 (Additional file 1: Table S6). Class-specific survival curves (A) and the inferred stage-specific (B) survival curves are shown in Fig. 2. The class 1 corresponds to multiple stages (i.e., IA, IB, IIA) and stage IIIA corresponds to multiple classes (i.e., 3, 4, 5), so the comparability is limited. Those who received surgery only (class 1) showed lower survival for the initial months, but higher long-term survival compared with those who received combined modality (class 2, 3, 4, 5). Within stage IIIA, those with neoadjuvant chemotherapy and surgery (class 5) showed long-term survival similar to that of stage IIB (class 2). When comparing the reference survival rates by the TNM classification, the class-specific survival rates based on the index algorithm under-estimated the survival probability for stage IIIB and IV, and over-estimated it for stage IIB (Table 3).

**Table 3:** Two-year and five-year survival by TNM classification (reference) vs index classification (N = 71,593)

TNM classification (reference)			Application of the index classification			
TNM Stage (7th ed.)	2-year survival^†^	5-year survival^†^	Classes in the proposed classification	N	Predicted 2-year survival (95% CI)	Predicted 5-year survival (95% CI)
IA	0.93	0.82	1	17,543	0.87 (0.86, 0.87)	0.79 (0.78, 0.79)
IB	0.85	0.66				
IIA	0.74	0.52				
IIB	0.64	0.47	2	6633	0.79 (0.78, 0.80)	0.54 (0.53, 0.55)
IIIA	0.55	0.36	3	1021	0.69 (0.66, 0.72)	0.35 (0.32, 0.38)
			4	999	0.64 (0.61, 0.67)	0.30 (0.27, 0.33)
			5	448	0.70 (0.65, 0.74)	0.47 (0.42, 0.51)
IIIB	0.34	0.19	6	21,378	0.26 (0.25, 0.26)	0.07 (0.06, 0.07)
IV	0.17	0.06	7	23,571	0.15 (0.15, 0.16)	0.04 (0.04, 0.04)

^†^Reference 2-year and 5-year survival probabilities are based on the IASLC Lung Cancer Staging Project (2016)

## Discussion

We proposed a stratification method using an inverse of the treatment decision rules that can classify lung cancer patients with real-world treatment records that are commonly available in routinely collected administrative databases. To validate the index classifier, we first evaluated the predictive performance of the index classifier against the reference standard staging information in an EHR database of a tertiary referral hospital. In addition, we examined whether the index classification showed differential survival rates when applied to a nationally representative administrative database that covers the hospital in the validation study. To our knowledge, this is the first study to apply and validate a stratification method based on an inverse of the treatment decision rules using both EHRs and a nationwide administrative database.

The index classification method considered treatment pattern-based categories as the baseline stratifying variable, rather than as a response or outcome variable. Stratifying phenotypes based on potential treatment options can be a practical way of defining patient categories. Modern classification of diseases started from Carl Linnaeus's classification (1707–1778), which influenced the international classification of disease and cause of death (ICD) [26, 27], which is an ontology that defines formal semantic relationships between concepts [27]. The TNM stage classification of cancer is a nomenclature describing the anatomic extent of cancer with limited predictive ability [28]. On the other hand, treatment decisions are made based on causal inference, or counterfactual prediction of an outcome given a treatment [29]. The classification of diseases has a major role in treatment decisions, but when its predictive ability is limited, additional qualitative clinical judgement is required [4, 28].

The index classification method used in the current study supplements the disease ontology information using both treatment information and treatment decision rules. The treatment decision rules provide the structural linkages between the disease information and the treatment information by the totality of the evidence in the literature. Utilization of treatment patterns enables the classification to be more interpretable and transparent to clinicians, and therefore, satisfies an essential need to deliver clinical impact with a data-driven system [30]. Treatment decision-making could also be seen as the ultimate goal of diagnostic development, which is denoted by the term "theragnostics" in certain fields [31, 32].

A practical advantage of the classification using an inverse of the treatment decision rules is that it can be applied to most routinely collected healthcare databases that have codified information of diagnoses, procedures, and treatments. Therefore, the classification can be applied to data generated while delivering care for building a “learning system” [33–35]. When the treatment decision rules are used for the baseline stratifying variable to be examined against future health outcomes, the results may feedback to update the treatment decision rules, enabling a continuous learning system. The index classification mitigates some known challenges in analyzing observational routinely-collected health data [12, 36, 37] by utilizing data for major procedures, such as surgery, chemotherapy, and radiotherapy [38]. Modeling the inverse of the decision function is a way of estimating the decision bases (i.e., patients' status, including stage and performance status) when the direct measurement is not available. This is in line with the estimation method of unmeasured decision bases using observed, or revealed, decision actions in economics [13] and statistics [14]. Treatment decisions can essentially encode such information, and therefore, it can be used as a proxy for such information [39], given a quantitative evaluation regarding the degree of bias and error. Therefore, it is an important first step to evaluate the predictive performance of the index classifier against the reference standard staging information in the EHRs.

Another advantage of the classification using an inverse of the treatment decision rules is that it may be considered as a phenotypical classification differentiating survival rates. In this sense, the treatment pattern for a patient can be considered as a decision function that maps from both the patient's performance status and the cancer stage into the treatment. Then, the survival prognosis from the patient's performance status and the cancer stage may be approximated by the classifier based on an inverse of the treatment decision rules. This approach may be useful when information on a patient's performance status is not readily available.

In the validation study using the EHRs, the index classifier showed high specificity for all stages of NSCLC. However, the sensitivity was particularly low for stage III (Table 2). There is especially high uncertainty when the treatment involves radiotherapy, which can be used for different purposes: it can be used as adjuvant or neoadjuvant therapy for surgical resection in early-stage disease, or it can be used as palliative treatment in combination with chemotherapy in advanced stage disease.

Differential class-specific survival rates have been used as a justification for the TNM classification of lung cancer [28, 40]. The methodological principles for development and validation of newer edition of the TNM classification are to make an ordinal classification system such that within-group variance is minimized while between-group variance is maximized, while not compromising the former edition. For lung cancer, the prognosis for survival was chosen as the measure for the clustering of the stage groups, while having the discriminatory power maintained across different factors, such as time, geographical location, diagnostic method, histology, and patient characteristics. Therefore, we examined whether the index classification results in differential class-specific survival rates in the underlying population. When the index classifier was applied to the nationwide administrative database, the class-specific survival rates based on the index algorithm under-estimated the survival probability for TNM stage IIIB and IV and over-estimated it for stage IIB. The discrepancies may rise from misclassifications of patients who received non-standard treatments due to unique clinical situations. The initially low survival of those who underwent surgery only (class 1) can be a result of immediate postoperative complications [41]. Observation of stage IIIA patients with neoadjuvant chemotherapy and surgery (class 5) showing long-term survival similar to stage IIB patients (class 2) is hypothesis-generating: this may be related to non-significant differences between adjuvant and neoadjuvant chemotherapy [42–44]. Additional studies need to confirm the long-term outcomes beyond five years among cancer survivors [45].

This study has several limitations. First, this study considered only a subdomain of treatment patterns that are invertible. When considering the decision functions with many-to-many relationships, inversion can be erroneous. The index classification showed some discrepancies compared to the TNM stage classification, and therefore, potential biases and errors should be addressed appropriately when it is used as a proxy variable. The present study was not meant to develop a precise prediction model, which needs to incorporate all important predictive factors, such as histology [46], genotype [47], environmental factors [48], and behavioral factors, such as smoking status [49]. Variations in healthcare practice need to be accounted for appropriately, and additional validation may be required before application to a new setting. Regional variations, such as the proportion of smokers [50, 51], genotype distribution [52, 53], and screening policy [54, 55], should also be considered when developing a precise prediction model. The utility of the index classification can be decreased in settings where treatment decision rules are less likely to be strictly followed, such as in budget-constrained settings.

Despite these limitations, the stratification of NSCLC patients using an inverse of the treatment decision rules can supplement a routinely collected database with information encoded in treatment decision rules to classify NSCLC patients by providing phenotypical classification that differentiates survival prognosis. Clinical prognostic value of the index classification needs to be validated and replicated in multiple clinical settings.

## Conclusion

We validated a stratification method of NSCLC patients using an inverse of the treatment decision rules in comparison to the TNM stage classification and demonstrated that the index classification has a potential to differentiate survival probabilities when applied to a population-based administrative database. The inverse of the treatment decision rules has a potential to supplement a routinely collected database with information encoded in treatment decision rules to classify NSCLC patients. It requires further validation and replication in multiple clinical settings.

## Supplementary Information

**Additional file 1**: **eTable 1**. A simplified summary of the treatment guideline used in this study. **eTable 2**. TNM staging of non-small cell lung cancer (7th edition). **eTable 3**. Confusion matrix comparing the true stage and the inferred stage in the validation study (N = 1375). **eTable 4**. Sensitivity analysis for the confusion matrix comparing the true stage and the inferred stage in the validation study, including the lung cancer patients with cardiopulmonary comorbidities (N = 1573). **eTable 5**. Sensitivity analysis for the stage-specific predictive performance metrics of the index classifier against the reference standard (TNM stage), including the lung cancer patients with cardiopulmonary comorbidity (N = 1573). **eTable 6**. Pairwise log-rank tests corrected with Bonferroni methods. **eMethods 1**. Explanation of the treatment guideline used in this study. **eMethods 2**. Rationale for the population selection. **eMethods 3**. Flow Diagram for the Analytic Steps in the Validation Study for the Index Classification using the Electronic Health Records. **eMethods 4**. Flow Diagram for the Analytic Steps for the Stage-specific Survival Analysis Applying the Index Classification in a Population-based Administrative Database.

## Data Availability

The data used in the validation study were provided by the Asan Medical Center (AMC). The data used in the application study were provided by the National Health Information Database (NHIS-2017-4-003) managed by the National Health Insurance Service (NHIS) of Korea. The data from AMC that are analysed during the current study are not publicly available due the institution's privacy regulations but are available from the corresponding author on reasonable request. The data from NHIS that are analysed during the current study are available from NHIS, but restrictions apply to the availability of these data, which were used under license for the current study, and so are not publicly available. Data are however available from the authors upon reasonable request and with permission of NHIS.
