# Supplementary material for: Stratifying non-small cell lung cancer patients using an inverse of the treatment decision rules: validation using electronic health records with application to an administrative database
Source: BMC Med Inform Decis Mak. 2023 Jan 6;23:3. doi: 10.1186/s12911-022-02088-x (PMC9825000; doi:10.1186/s12911-022-02088-x)
Supplement: Supplementary file 1 — Additional file 1: eTable 1. A simplified summary of the treatment guideline used in this study. eTable 2. TNM staging of non-small cell lung cancer (7th edition). eTable 3. Confusion matrix comparing the true stage and the inferred stage in the validation study (N = 1375). eTable 4. Sensitivity analysis for the confusion matrix comparing the true stage and the inferred stage in the validation study, including the lung cancer patients with cardiopulmonary comorbidities (N = 1573). eTable 5. Sensitivity analysis for the stage-specific predictive performance metrics of the index classifier against the reference standard (TNM stage), including the lung cancer patients with cardiopulmonary comorbidity (N = 1573). eTable 6. Pairwise log-rank tests corrected with Bonferroni methods. eMethods 1. Explanation of the treatment guideline used in this study. eMethods 2. Rationale for the population selection. eMethods 3. Flow Diagram for the Analytic Steps in the Validation Study for the Index Classification using the Electronic Health Records. eMethods 4. Flow Diagram for the Analytic Steps for the Stage-specific Survival Analysis Applying the Index Classification in a Population-based Administrative Database. [file 12911_2022_2088_MOESM1_ESM.pdf]

<*Supplementary Materials*>

Stratifying non-small cell lung cancer patients using an inverse of the treatment decision rules - validation using electronic health records with application to an administrative database

Authors

Min-Hyung Kim, MD, MS[1, 2*]; Sojung Park, MD[3*]; Yu Rang Park, PhD[4]; Wonjun Ji, MD[5]; Seul-gi Kim, MA[5]; Minji Choo, RN[5, 6]; Seung-Sik Hwang, MD, PhD[6]; Jae Cheol Lee, MD, PhD[7]; Hyeong Ryul Kim, MD, PhD[8]; Chang-Min Choi, MD, PhD[5, 7]

* Min-Hyung Kim and Sojung Park contributed equally as co-first authors.

Author Affiliations:

[1] Department of Epidemiology, Harvard T.H. Chan School of Public Health, Harvard University, Boston, MA, USA
[2] Department of Biomedical Sciences, College of Medicine, Seoul National University, Seoul, Republic of Korea
[3] Department of Respiratory and Critical Care Medicine, College of Medicine, Ewha Womans University Medical Center, Seoul, Republic of Korea
[4] Department of Biomedical System Informatics, Yonsei University College of Medicine, Seoul, Republic of Korea
[5] Department of Respiratory and Critical Care Medicine, University of Ulsan College of Medicine, Asan Medical Center, Seoul, Republic of Korea
[6] Department of Epidemiology, Graduate School of Public Health, Seoul National University, Seoul, Republic of Korea
[7] Department of Oncology, University of Ulsan, College of Medicine, Asan Medical Center, Seoul, Republic of Korea
[8] Department of Thoracic and Cardiovascular Surgery, University of Ulsan, College of Medicine, Asan Medical Center, Seoul, Republic of Korea

Contact Information of the Corresponding Author
Chang-Min Choi, MD, PhD
Department of Pulmonary and Critical Care Medicine
Department of Oncology
Asan Medical Center, University of Ulsan College of Medicine, 88 Olympic-ro 43-gil, Songpa–gu, Seoul, 05505, South Korea
E-mail: ccm@amc.seoul.kr
Tel: +82) 2–3010–5902
Fax: +82) 2–3010–6968

Table of Contents

eTable 1. A simplified summary of the treatment guideline used in this study.† 3

eTable 2. TNM staging of non-small cell lung cancer (7th edition). 4

eTable 3. Confusion matrix comparing the true stage and the inferred stage in the validation study (N = 1,375). 5

eTable 4. Sensitivity analysis for the confusion matrix comparing the true stage and the inferred stage in the validation study, including the lung cancer patients with cardiopulmonary comorbidities (N = 1,573). 5

eTable 5. Sensitivity analysis for the stage-specific predictive performance metrics of the index classifier against the reference standard (TNM stage), including the lung cancer patients with cardiopulmonary comorbidity (N = 1,573). 5

eTable 6. Pairwise log-rank tests corrected with Bonferroni methods. 6

eMethods 1. Explanation of the treatment guideline used in this study. 7

eMethods 2. Rationale for the population selection. 10

eMethods 3. Flow Diagram for the Analytic Steps in the Validation Study for the Index Classification using the Electronic Health Records. 11

eMethods 4. Flow Diagram for the Analytic Steps for the Stage-specific Survival Analysis Applying the Index Classification in a Population-based Administrative Database. 12

eTable 1. A simplified summary of the treatment guideline used in this study.†

TNM 7th	Treatment		
IA, IB†	Surgical resection		
IIA, IIB	Surgical resection	Adjuvant CTx	
IIIA‡	Surgical resection	Adjuvant CTx	Adjuvant RTx
	Surgical resection	Adjuvant RTx	Adjuvant CTx
	Neoadjuvant CTx	Surgical resection	
IIIA, IIIB	CCRT		
IV	CTx		

CTx, chemotheraphy; RTx, radiation therapy; CCRT, concurrent chemoradiation therapy.

† See eMethods 1 for further explanation of the treatment guideline.

‡ Stage III A patients have multiple treatment options with multiple scenarios. See eMethods 1 for further explanation.

eTable 2. TNM staging of non-small cell lung cancer (7th edition).

TNM descriptors	N0	N1	N2	N3
T1a	I A	II A	III A	III B
T1b	I A	II A	III A	III B
T2a	I B	II A	III A	III B
T2b	II A	II B	III A	III B
T3	II B	III A	III A	III B
T4	III A	III A	III B	III B
M1a	IV	IV	IV	IV
M1b	IV	IV	IV	IV

* TNM descriptors for lung cancer (7th edition)

T: Tumour

TX	Primary tumour cannot be assessed, or tumour proven by the presence of malignant cells in sputum or bronchial washings but not visualized by imaging or bronchoscopy
T0	No evidence of primary tumour
Tis	Carcinoma in situ
T1	Tumour < 3 cm in greatest dimension, surrounded by lung or visceral pleura, without bronchoscopic evidence of invasion more proximal than the lobar bronchus (i.e., not in the main bronchus)
T1a	Tumour < 2 cm in greatest dimension
T1b	Tumour > 2 cm but < 3 cm in greatest dimension
T2	Tumour > 3 cm but < 7 cm or tumour with any of the following features (T2 tumours with these features are classified T2a if < 5 cm):
	Involves main bronchus, > 2 cm distal to the carina
	Invades visceral pleura
	Associated with atelectasis or obstructive pneumonitis that extends to the hilar region but does not involve the entire lung
T2a	Tumour > 3 cm but < 5 cm in greatest dimension
T2b	Tumour > 5 cm but < 7 cm in greatest dimension
T3	Tumour > 7 cm or one that directly invades any of the following:
	Chest wall (including superior sulcus tumours), diaphragm, phrenic nerve, mediastinal pleura, parietal pericardium
	Tumour in the main bronchus < 2 cm distal to the carina but without involvement of the carina
	Associated atelectasis or obstructive pneumonitis of the entire lung
	Separate tumour nodule(s) in the same lobe
T4	Tumour of any size that invades any of the following:
	Mediastinum, heart, great vessels, trachea, recurrent laryngeal nerve, esophagus, vertebral body, carina
	Separate tumour nodule(s) in a different ipsilateral lobe

N: Nodes

NX	Regional lymph nodes cannot be assessed
N0	No regional lymph node metastasis
N1	Metastasis in ipsilateral peribronchial and/or ipsilateral hilar lymph nodes and intrapulmonary nodes, including involvement by direct extension
N2	Metastasis in ipsilateral mediastinal and/or subcarinal lymph node(s)
N3	Metastasis in contralateral mediastinal, contralateral hilar, ipsilateral or contralateral scalene, or supraclavicular lymph node(s)

M: Metastases

MX	Distant metastasis cannot be assessed
M0	No distant metastasis
M1	Distant metastasis
M1a	Separate tumour nodule(s) in a contralateral lobe tumour with pleural nodules or malignant pleural/ pericardial effusion
M1b	Distant metastasis

eTable 3. Confusion matrix comparing the true stage and the inferred stage in the validation study (N = 1,375).

True Stage	Inferred Stage				
	I	II	III	IV	Total
I	0.679	0.007	0.000	0.000	0.686
II	0.013	0.073	0.002	0.001	0.089
III	0.001	0.010	0.051	0.017	0.079
IV	0.003	0.009	0.000	0.134	0.145
Total	0.697	0.099	0.053	0.151	1.000

eTable 4. Sensitivity analysis for the confusion matrix comparing the true stage and the inferred stage in the validation study, including the lung cancer patients with cardiopulmonary comorbidities (N = 1,573).

True Stage	Inferred Stage				
	I	II	III	IV	Total
I	68.2%	0.6%	0.1%	0.0%	68.8%
II	1.4%	7.4%	0.3%	0.1%	9.1%
III	0.2%	1.1%	5.0%	1.8%	8.1%
IV	0.3%	0.8%	0.0%	12.9%	14.0%
Total	70.1%	9.9%	5.3%	14.7%	100.0%

eTable 5. Sensitivity analysis for the stage-specific predictive performance metrics of the index classifier against the reference standard (TNM stage), including the lung cancer patients with cardiopulmonary comorbidity (N = 1,573).

Metric	I	II	III	IV	Average
Sensitivity	0.99 (0.99, 0.99)	0.81 (0.74, 0.87)	0.62 (0.54, 0.70)	0.92 (0.89, 0.96)	0.84 (0.81, 0.86)
Specificity	0.94 (0.92, 0.96)	0.97 (0.96, 0.98)	0.99 (0.99, 0.99)	0.98 (0.97, 0.99)	0.97 (0.97, 0.98)
Positive Predictive Value	0.97 (0.96, 0.98)	0.75 (0.68, 0.82)	0.94 (0.89, 0.99)	0.88 (0.83, 0.92)	0.88 (0.86, 0.91)
Negative Predictive Value	0.98 (0.96, 0.99)	0.98 (0.97, 0.99)	0.97 (0.96, 0.98)	0.99 (0.98, 0.99)	0.98 (0.97, 0.98)
F1 score	0.98 (0.98, 0.99)	0.78 (0.72, 0.83)	0.75 (0.68, 0.81)	0.90 (0.87, 0.92)	0.85 (0.82, 0.88)
c-statistic	0.97 (0.95, 0.98)	0.89 (0.86, 0.92)	0.81 (0.77, 0.85)	0.95 (0.93, 0.97)	0.90 (0.89, 0.92)

eTable 6. Pairwise log-rank tests corrected with Bonferroni methods.

	Class 1	Class 2	Class 3	Class 4	Class 5	Class 6
Class 2	< 0.001					
Class 3	< 0.001	< 0.001				
Class 4	< 0.001	< 0.001	0.86			
Class 5	< 0.001	0.62	0.002	< 0.001		
Class 6	< 0.001	< 0.001	< 0.001	< 0.001	< 0.001	
Class 7	< 0.001	< 0.001	< 0.001	< 0.001	< 0.001	< 0.001

eMethods 1. Explanation of the treatment guideline used in this study.

- According to the treatment guidelines for NSCLC, chemotherapeutic agents were identified with medication codes, and surgical resection and radiotherapy were identified with procedural codes for Korean National Health Insurance Services (KNHIS). Surgical resection included wedge resection, segmentectomy, lobectomy, bilobectomy, lobectomy combined with wedge resection, sleeve lobectomy, pneumonectomy, and sleeve pneumonectomy.
- The treatment guideline used in this study is based on Ettinger DS, Bepler G, Bueno R, et al. Non-small cell lung cancer: Clinical Practice Guidelines in Oncology. J Natl Compr Cancer Netw. 2006;4(6):548–582.
- Stage I
    - If the patient is operable, treat with surgery alone.
    - If the patient cannot undergo a surgical operation, then treat with radiotherapy alone.
        - The patient is inoperable if (1) the pulmonary function is very poor so that the patient is not likely to survive with the remaining lung after the resection, or if (2) general condition is very poor so that the patient is not likely to endure the anesthesia and surgical procedure.
- Stage II
    - Perform surgical resection and adjuvant chemotherapy.
        - The regimen for adjuvant chemotherapy is cisplatin/or carboplatin + vinorelbine/or paclitaxel.
    - If the size is 3cm or larger but there is no lymph node invasion and no visceral pleura invasion, surgery alone without adjuvant chemotherapy can be performed. However, this is a rare scenario.
    - Stage II patients may receive surgical resection only because of poor general status or patient's refusal. This may result in a misclassification of Stage II patients with Stage I patients when using the index classification method.
- Stage III
    - NSCLC Stage III is a condition with a large tumor size with an invasion to a major organ or ipsilateral/contralateral mediastinal lymph node invasion. Therefore, this condition has multiple scenarios with multiple treatment options.
        - According to the NCCN guideline, the treatment decision for NSCLC Stage III is based on the tumor location, pulmonary function, and comorbid conditions.
        - For NSCLC Stage III, the treatment decisions for surgery, radiotherapy, and chemotherapy are individualized.
        - A multidisciplinary clinic that coordinates the care among medical, radiation, and surgical oncology is particularly effective for NSCLC stage III (Friedman et al. J Multidiscip Healthc. 2016. PMID: 27358568).
        - It has been reported that curative-intent treatments were less frequently applied to elderly patients particularly in NSCLC stage III (Driessen et al. Lung Cancer. 2018. PMID: 29413051).
        - The treatment trend for NSCLC stage III is also rapidly changing, including neoadjuvant chemotherapy, radiotherapy, and immunotherapy (Makimoto G, et al. Respir Investig. 2019. PMID 31000495).
    - If the lesion is large but distant from major organs, and the lesion is anatomically resectable with a surgical operation, surgery is performed first and then additional adjuvant chemotherapy and adjuvant radiotherapy are performed.
        - The regimen for adjuvant chemotherapy is cisplatin/or carboplatin + vinorelbine/or paclitaxel.
    - If the lesion is expected to be anatomically resectable if the size is reduced after neoadjuvant chemotherapy, neoadjuvant chemotherapy is performed first and then a surgical operation is performed.

        - The regimen for neoadjuvant chemotherapy is cisplatin/or carboplatin + vinorelbine/or paclitaxel.
    - For the patient with Stage III B with a lesion that is not resectable, concurrent chemotherapy and radiotherapy is performed.
        - The regimen for concurrent chemoradiation therapy is cisplatin/or carboplatin + paclitaxel/or etoposide.
    - If the lymph node invasion is only in the ipsilateral peribronchial and/or ipsilateral hilar lymph nodes and intrapulmonary nodes, including involvement by direct extension (N1), without any mediastinal lymph nodes, surgical resection and adjuvant chemotherapy can be performed without adjuvant radiotherapy. This may result in a misclassification of Stage III patients with Stage II patients when using the index classification method.
    - If the lesion involves the scalene or the brachial plexus, chemotherapy and radiotherapy may be performed before surgery to avoid resection of nerves.
    - If contralateral lymph nodes are invaded, surgery is not performed because of the risk of resecting both lungs. In this case concurrent chemoradiation therapy alone is performed.
- Stage IV
    - If the patient has an *EGFR* mutation, treat with gefitinib, erlotinib, or afatinib.
    - If the patient has an *ALK* translocation, treat with crizotinib.
    - If the patient has no mutation, treat with cisplatin/or carboplatin + pemetrexed/ or paclitaxel/ or etoposide/ or docetaxel/ or gemcitabine/ or vinorelbine.
    - During the study period, immunotherapy was not available.
- Treatment options for special cases
    - Radiotherapy alone may be performed in many different scenarios. Stage I patients may receive radiotherapy alone when they are not operable. Stage IV patients may receive radiotherapy alone for a palliative purpose. This treatment option was not considered in this study due to the uncertainty of the purpose of the treatment.
    - Surgery with postoperative radiotherapy alone may be performed if the surgery failed to resect the lesion completely or if there is a suspicion for a recurrence. This treatment option was not considered in this study due to the uncertainty of the purpose of the treatment.
    - Concurrent chemoradiation therapy and surgical resection may be performed when the lesion is around a vital organ, such as the heart. This treatment option was not considered in this study due to the rarity of the condition.
    - Neoadjuvant chemotherapy and surgery with postoperative radiotherapy may be performed when the surgery fails to resect the lesion completely. This treatment option was not considered in this study due to the rarity of the condition.
    - If the patient has a poor performance status and the lesion is operable and resectable, perform the surgery and omit the chemotherapy and radiotherapy. This may result in an underestimation of the patients' stage when using the index classification method.
    - If the patient has a poor performance status and the lesion is not operable or not resectable, perform the radiotherapy and omit the chemotherapy. If the patient cannot even tolerate the radiotherapy, just provide the best supportive care. This treatment option was not considered in this study due to the uncertainty of the purpose of the treatment.
- Surgical options
    - The first choice is lobectomy.
    - However, if the lung function is poor or there is a ground glass opacity, wedge resection or segmentectomy can be considered.
    - If the lesions are located in two or more lobes, bilobectomy is performed.

    - If the lesion involves the main bronchus, pneumonectomy is performed. Pneumonectomy is more likely to happen in stage III. However, even in stage III, the most common surgical choice is lobectomy.
- Chemotherapy indications
    - Cytotoxic chemotherapeutic agents are recommended in Eastern Cooperative Oncology Group (ECOG) grade 0 or 1.
        - ECOG grade 0: Fully active, able to carry on all pre-disease performance without restriction
        - ECOG grade 1: Restricted in physically strenuous activity but ambulatory and able to carry out work of a light or sedentary nature, e.g., light house work, office work
    - Chemotherapy is less likely to be used in elderly patients (Driessen EJM, et al. Lung Cancer 2018).
    - Chemotherapy is less likely to be used in patients with major comorbidities.
    - If the patient is inoperable, chemotherapy and radiotherapy can be considered as alternatives.

eMethods 2. Rationale for the population selection.

- Exclusion of the patients initially diagnosed at an external hospital: the diagnostic information from the external hospital may be incomplete, not in the same standards as the study hospital, or not stored in a readily accessible format (e.g., scanned fax files).
- Exclusion of the patients transferred to an external hospital: the diagnostic information may be incomplete, and the treatment information may not be available.
- Lung cancer stage missing or erroneous: incomplete information to validate the index classification.
- No treatment: cannot apply the index classification based on the treatment information.
- Age less than 20: the patient is treated by different treatment teams (e.g., pediatric oncology, pediatric surgery).
- Age greater than or equal to 75: chemotherapy is less likely to be used in elderly patients regardless of ECOG grade (Driessen EJM, et al. Lung Cancer 2018).
- Age missing: excluded because the age may be less than 20 or greater than or equal to 75.
- Patients with preexisting non-pulmonary cancer: second primary cancers have different treatment guidelines.
- Patients with cardiopulmonary comorbidity: cardiopulmonary comorbidity can limit major treatments.

eMethods 3. Flow Diagram for the Analytic Steps in the Validation Study for the Index Classification using the Electronic Health Records.

1. Estimation of sample size: 108 for each category for testing the accuracy of a single modality to detect a pre-specified area under the receiver operating characteristic curve value of 0.8 against a null value of 0.7 at the 95% confidence level and 80% power.

▼

2. Identification of study population based on the inclusion criteria: newly diagnosed with NSCLC stage between 2011 and 2015, covered under the national health insurance, and received cancer treatment in the hospital, with the last visit within 180 days from the end of the observation period in the EHR database.

▼

3. Application of the exclusion criteria: initially diagnosed at an external hospital, transferred to an external hospital, missing stage, erroneous stage identified during the manual chart review process, received no treatment, missing age information, age less than 20 years or more than 75 years, preexisting non-pulmonary cancer, or cardiopulmonary comorbidity.

▼

4. Application of the index classification method.

▼

5. Calculation of performance metrics: overall accuracy is calculated as the proportion of correctly classified stages among the entire study population. Stage-specific sensitivity, specificity, positive predictive value, negative predictive value, and c-statistic were calculated by contrasting each stage with alternative stages.

▼

6. Calculation of the confidence interval of the performance metrics: confidence intervals for the overall accuracy, stage-specific sensitivity, specificity, positive predictive value, negative predictive value, and c-statistic were calculated based on the 1000 bootstrap resamples.

eMethods 4. Flow Diagram for the Analytic Steps for the Stage-specific Survival Analysis Applying the Index Classification in a Population-based Administrative Database.

1. Identification of study population based on the inclusion criteria: patients with a new diagnosis code for lung cancer with at least one claim code for the treatment of NSCLC between 2004 and 2013, among those without any diagnosis code for lung cancer between 2002 and 2003 (washout period).

▼

2. Application of the exclusion criteria: received no treatment, age less than 20 years or more than 75 years, preexisting non-pulmonary cancer, or cardiopulmonary comorbidity.

▼

3. Application of the index classification method.

▼

4. Proportional hazard assumption rejected: Schoenfeld residuals showed a non-random pattern with significant dependency with time.

▼

5. The survival function was estimated with the non-parametric Kaplan-Meier method, and the log-rank test was used to test the null hypothesis of no difference in the survival between groups.

▼

6. Pairwise log-rank tests corrected with Bonferroni methods were performed to explore significantly different survival curves.
